# SAA1 identified as a potential prediction biomarker for metastasis of hepatocellular carcinoma *via* multi-omics approaches

**DOI:** 10.3389/fonc.2023.1138995

**Published:** 2023-04-04

**Authors:** Gang Li, Qingrong Shen, Haotian Xu, Ying Zhou, Cuiping Li, Yasi Li, Min He

**Affiliations:** ^1^ School of Public Health, Guangxi Medical University, Nanning, China; ^2^ Department of Pharmacy, The People’s Hospital of Guangxi Zhuang Autonomous Region, Nanning, China; ^3^ The First Affiliated Hospital, Guangxi Medical University, Nanning, China; ^4^ School of Stomatology, Guangxi Medical University, Nanning, China; ^5^ Department of Public Health Sciences, College of Medicine, Pennsylvania State University, Hershey, PA, United States; ^6^ Laboratory Animal Center, Guangxi Medical University, Nanning, China; ^7^ Key Laboratory of High-Incidence-Tumor Prevention and Treatment, Guangxi Medical University, Ministry of Education, Nanning, China

**Keywords:** SAA1, hepatocellular carcinoma, metastasis, biomarker, multi-omics, bioinformatics

## Abstract

**Background:**

Metastasis is the major cause of high recurrence and mortality of hepatocellular carcinoma (HCC). Unfortunately, there are few reports on effective biomarkers of HCC metastasis. Previous studies have reported that SAA1 may be a predictor and prognostic biomarker for multiple malignant tumors. However, the role of SAA1 in HCC has not yet been investigated.

**Methods:**

We applied RNA sequencing and proteomics analysis to investigate the expression landscape of HCC cell lines and patient serum, respectively. SAA1 is a common key gene and listed as a candidate biomarker of HCC metastasis. It was validated in two cell lines, 107 participants serum, and 63 matched HCC and adjacent non-tumorous liver tissues. Human Protein Atlas (HPA), Genotype-Tissue Expression (GTEx), and The Cancer Genome Atlas (TCGA) datasets were integrated to explore SAA1 expression among various cell types and organs. The diagnostic and prognostic value of SAA1 in HCC were determined through receiver operating characteristic (ROC) and Kaplan–Meier curves. Gene Ontology (GO), Kyoto Encyclopedia of Genes and Genomes (KEGG) enrichment analysis, and protein-protein interaction (PPI) network were constructed for SAA1, as well as for its co−expressed genes. We further analyzed the correlation between SAA1 and co-expression genes.

**Results:**

We found 7 differentially expressed genes (DEGs) and 14 differentially expressed proteins (DEPs) were related to HCC metastasis. SAA1, a key candidate biomarker, was highly enriched in hepatocytes and liver organ, and it was also highly expressed in HCC cells and the serum and tissues of HCC patients. The results of ROC curve analysis indicated that SAA1 had better predictive values for distinguishing HCC metastasis from non-metastasis. Kaplan-Meier curve analysis revealed that HCC patients with higher SAA1 expression had worse overall survival.

**Conclusions:**

Our findings provide new insights into HCC metastasis by identifying candidate gene prediction biomarkers for HCC metastasis.

## 1 Introduction

Hepatocellular carcinoma (HCC) is one of the most frequently diagnosed malignancies and the fourth leading cause of cancer-related deaths (1, 2). Chronic infection with hepatitis B virus (HBV) or hepatitis C virus (HCV) is the leading cause of HCC, and it mostly occurs in countries and regions with low economic development (3). Tumor metastasis is still a major cause of high recurrence and mortality of HCC. Although there are some effective treatments, the overall prognosis of HCC remains extremely poor, with a 5-years survival rate only about 18% (4). Therefore, it is urgent to elucidate the molecular mechanism of HCC metastasis and identify potential molecular biomarkers for prognosis prediction.

In recent decades, the advancements and availability of multi-omics technologies, including genomic/transcriptomic sequencings and proteomic/metabolomic mass spectra, are making it possible for more informative biomarkers to be identified and developed for clinical practice (5). Consequently, multi-omics technologies have been widely applied to analyze liquid- and tissue-derived samples from HCC patients. The integration of multi-omics data has significantly improved our knowledge in a variety of diseases, especially the discovery of valuable HCC biomarkers. Although there are some existing biomarkers for HCC, limited number of reports on biomarkers of HCC metastasis.

SAA1 (serum amyloid A-1 protein), as a member of the serum amyloid A family of apolipoproteins, is a sensitive acute phase high-density lipoprotein. SAA1 plays a critical role in cholesterol homeostasis and high-density lipoprotein metabolism (6, 7). SAA1 expression is upregulated when the body is stressed by inflammation and tissue damage (8). At the same time, it participates in the body’s immune system by facilitating the repairment of injured tissues and used as a diagnostic or prognostic marker for many diseases (9, 10). In recent years, the role of SAA1 in the occurrence and development of tumors has received increasing attention (11–19). Previous literatures have reported that SAA1 could contribute to cancer development and accelerate tumor progression and distant metastasis (20). Moreover, the up-regulation of SAA1 could be used as a predictor and prognostic biomarker for a variety of malignant tumors (21–24). For example, SAA1 produced by colorectal cancer cells recruits neutrophils to the invasive front of colorectal cancer, and stimulates neutrophils to produce CXCL8 and MMP-9, which contribute to tumor progression (25). In addition, tumor‐associated macrophages promote aggressive behavior by colorectal cancer cells through upregulation of SAA1 *via* IL‐1β signaling (26). However, the diagnostic and prognostic potential of SAA1 in HCC has not yet been investigated.

In this study, by integration of multi-omics data such as transcriptomic and proteomic, we found that SAA1 expression was significantly up-regulated in HCC and may be associated with HCC metastasis. Subsequently, SAA1 expression was validated in HCC cells and HCC patient serum and tissues. Furthermore, the predictive value of SAA1 was evaluated using the receiver operating characteristic (ROC) curve and survival analysis. We found that SAA1 had great potential to be a prediction biomarker for HCC metastasis, which will shine a light on HCC metastasis.

## 2 Materials and methods

### 2.1 Patients

A total of 63 patients who were diagnosed with HCC and treated with partial liver resection surgery at the Affiliated Tumor Hospital of Guangxi Medical University were enrolled, including 52 males and 11 females, with a mean age of 47.86 years (range 28 to 71 years). The patients were pathologically diagnosed with HCC of histological grade II (n = 28), grade III (n = 20), and grade IV (n = 15) according to the modified nuclear grading scheme outlined by the Edmondson and Steiner system. The inclusion criteria were as follows (1): histologically confirmed or image-diagnosed primary HCC; (2) aged 18 years or older; (3) absence of macrovascular invasion or extrahepatic disease. The exclusion criteria were as follows: (1) with other malignancies; (2) receiving other antitumor treatments; (3) lost to follow-up; (4) incomplete clinical data. A summary of patient characteristics and pathological characteristics is presented in **Table 1**. This study was approved by the Ethics Committee of Guangxi Medical University. All patients provided written informed consent to participate in this study.

**Table 1:** Clinicopathological variables of 63 patients with hepatocellular carcinoma (HCC).

Variables	n = 63
Age (years)	
≤ 50	36
> 50	27
Gender	
Male	52
Female	11
Serum AFP (ng/ml)	
≤ 25	28
25 - 400	6
> 400	29
Tumor size (cm)	
≤ 10	46
> 10	17
Portal vein thrombosis	
Presence	20
Absence	43
Diolame complete	
Yes	28
No	35
Tumor nodule number	
Solitary	44
Multiple (≥2)	19
Edmondson grade	
II	28
III	20
IV	15
Liver cirrhosis	
Yes	53
No	10
Hepatitis B virus DNA	
Positive	42
Negative	21
Recurrence	
Yes	32
No	31

### 2.2 Cell lines and tissue samples

The human HCC cell line SMMC-7721 and primary human normal liver cell line HL-7702 were purchased from the Committee on Type Culture Collection of Chinese Academy of Sciences (Shanghai, China). All cell lines were maintained under recommended culture conditions. Cells were incubated in a 37°C humidified incubator containing 5% CO_2_. All HCC tissues and matched adjacent non-tumorous liver tissues were obtained immediately after hepatectomy and frozen in liquid nitrogen and stored at -80°C, or collected in 10% formalin and embedded in paraffin for histopathological analysis.

### 2.3 Serum samples

Sera for proteomics analysis was obtained from a total of 20 participants at the First Affiliated Hospital of Guangxi Medical University between 2009 and 2012. Ten HCC patients were comprehensively diagnosed *via* imaging studies, serology, and pathological examination without radiation or chemotherapy treatment. Ten healthy age- and gender-matched donors served as controls. All serum was obtained from fasting blood and collected into serum separation tubes. Serum samples for enzyme-linked immunosorbent assay (ELISA) were taken from 107 participants at the Affiliated Cancer Hospital of Guangxi Medical University between 2011 and 2012. A total of 55 patients were diagnosed with HCC based on computed tomography, magnetic resonance imaging, and histopathological findings; 13 of them were diagnosed with HCC metastasis (10 lung metastasis and 3 lymph metastasis). Sera from 52 healthy donors was used as a control. All HCC and controls subjects were from the same geographic region (Guangxi Zhuang Autonomous Region, China) and ethnic origin (Han ethnicity). Detailed patient and donor information is summarized in **Table 2**. All subjects included in this study provided written informed consent, and the protocols conducted in accordance with guidelines outlined by the Ethics Committee of Guangxi Medical University.

**Table 2:** Detailed summary of serum samples.

Group	n	Sex (M/F)	Mean ± SD			
			Age	AFP (ng/mL)^3^	ALT (IU/L)^3^	AST (IU/L)^3^
Group 1 ^1^						
Control	10	9/1	55.20 ± 10.73	negative	negative	negative
HCC	10	9/1	53.25 ± 9.78	319.22 ± 106.03	83.11 ± 92.58	121.56 ± 155.61
Group 2 ^1^						
Control	52	34/18	51.25 ± 13.75	negative	negative	negative
HCC ^2^	55	36/19	46.23 ± 11.34	514.37 ± 205.04	95.45 ± 70.23	117.68 ± 123.74
HCCM ^2^	13	9/4	49.25 ± 10.17	651.64 ± 301.59	93.79 ± 69.41	120.95 ± 113.28

^1^Group 1, samples for proteomics analysis; Group 2, samples for enzyme-linked immunosorbent assay (ELISA).

^2^HCC, hepatocellular carcinoma; HCCM, HCC with metastasis.

^3^AFP, alpha-fetoprotein; ALT, alanine transaminase; AST, aspartate aminotransferase.

### 2.4 Transcriptomic sequencing

RNA was extracted from HL-7702 and SMMC-7721 cells using TRIzol reagent kit (Invitrogen, MA, USA) according to the manufacturer’s protocol. mRNA was purified using oligo-dT beads from 100μg of total RNAs for each sample and then fragmented. The cleaved RNA fragments were reverse-transcribed into first-strand cDNA, followed by second-strand cDNA synthesis. Next, a single ‘A’ base was added to the cDNA fragments at the 3’ end. The cDNAs were ligated to adapters and enriched by polymerase chain reaction (PCR) to generate the final cDNA library. After amplifying the sequencing template, RNA sequencing was performed using the Ion Proton System (Life Technologies, MA, USA) with the standard protocol. A schematic of the transcriptomic sequencing process is shown in **Supplemental Figure 1**.

### 2.5 Proteomics analysis

The serum samples (5μl/subject) from HCC and control groups were pooled (n = 10 subjects/group), diluted thrice, and transferred to a spin filter to remove particles. The highly abundant proteins were discarded with Agilent Human 14 Multiple Affinity Removal System Column (Agilent Technologies, Waldron, Germany). The sample was digested by pancreatin and labeled with 8-plex isobaric tags for relative and absolute quantitation (iTRAQ) reagent kit (Applied Biosystems, CA, USA). The peptides were separated by strong cation exchange (SCX) chromatography through Tempo LC MALDI Spotting system (Applied Biosystems, CA, USA) and processed by 5800 MALDI-Time-of-Flight (TOF)/TOF mass spectrometer (Applied Biosystems, CA, USA). Proteins were identified against the International Protein Index (IPI) database (ipi.HUMAN.V3.62.fasta). A flow chart of iTRAQ-MALDI-MS/MS analysis is shown in **Supplemental Figure 2**.

### 2.6 RNA extraction and qRT-PCR

Total RNA in cells was extracted in accordance with the manual provided with the TRIzol reagent (Invitrogen, MA, USA). The extracted total RNA was treated with RNase-free DNase (Invitrogen, MA, USA) and quantified by NanoDrop 2000 (Thermo-Fisher Scientific, MA, USA). The reverse transcription of mRNAs was performed in accordance with the manual provided by M-MLV First Strand Kit (Invitrogen, MA, USA). Fluorescence quantitative PCR was performed in accordance with the manual provided by the FastStart Universal SYBR Green Master (Roche, Shanghai, China).

### 2.7 ELISA

The level of SAA1 protein in sera was measured using a Human SAA sandwich ELISA kit (CUSABIO, China). Serum samples were assayed after 10-fold dilution. This test was run twice according to the manufacturer’s protocol.

### 2.8 Immunohistochemistry and immunohistochemical assessment

Immunohistochemical staining (IHC) were performed on formalin-fixed, paraffin-embedded tissue sections obtained from HCC patients according to standard procedures. The sections were blocked for 30 min using 10% normal goat serum, and they were separately incubated with the primary antibodies directed against SAA1 at 37°C for 3 hours. After washing, sections were incubated for 30 min with biotinylated secondary antibody (Envision™ Detection Kit; Gene Tech, Shanghai, China) at 37°C. The staining of sections was performed using the streptavidin-biotin-peroxidase complex for SAA1. The complex was visualized with diaminobenzidine (DAB) and counterstained with hematoxylin. The sections were then dehydrated in a graded series of alcohol, cleared in xylene, and mounted onto glass slides. The staining was quantified by digital image analysis with Image-Pro Plus 6.0 software (Media Cybernetics, MD, USA).

### 2.9 Bioinformatics analyses

The protein-protein interaction (PPI) network of differentially expressed proteins (DEPs) was acquired from the Search Tool for Retrieval of Interacting Genes/Proteins (STRING) database (version 11.0). Validation of SAA1 based on Human Protein Atlas (HPA) database and Genotype-Tissue Expression (GTEx) database. Gene Ontology (GO) and Kyoto Encyclopedia of Genes and Genomes (KEGG) pathways analysis were performed. Correlation analysis between SAA1 expression level and co-expression genes in HCC based on Tumor Immune Estimation Resource (TIMER) database.

### 2.10 Statistical analysis

SPSS Statistics 20.0 (IBM Corp., NY, USA) and Graphpad Prism 8.0.1 (GraphPad Software, CA, USA) were used for statistical analysis. The ROC curve was used to distinguish HCC patients and obtain the area under the curve (AUC). Data was presented as mean ± SD. Data were considered statistically significant as follows: *P < 0.05, **P < 0.01, and ***P < 0.001.

## 3 Results

### 3.1 Overview of transcriptomic and proteomic analysis

**Figure 1:**
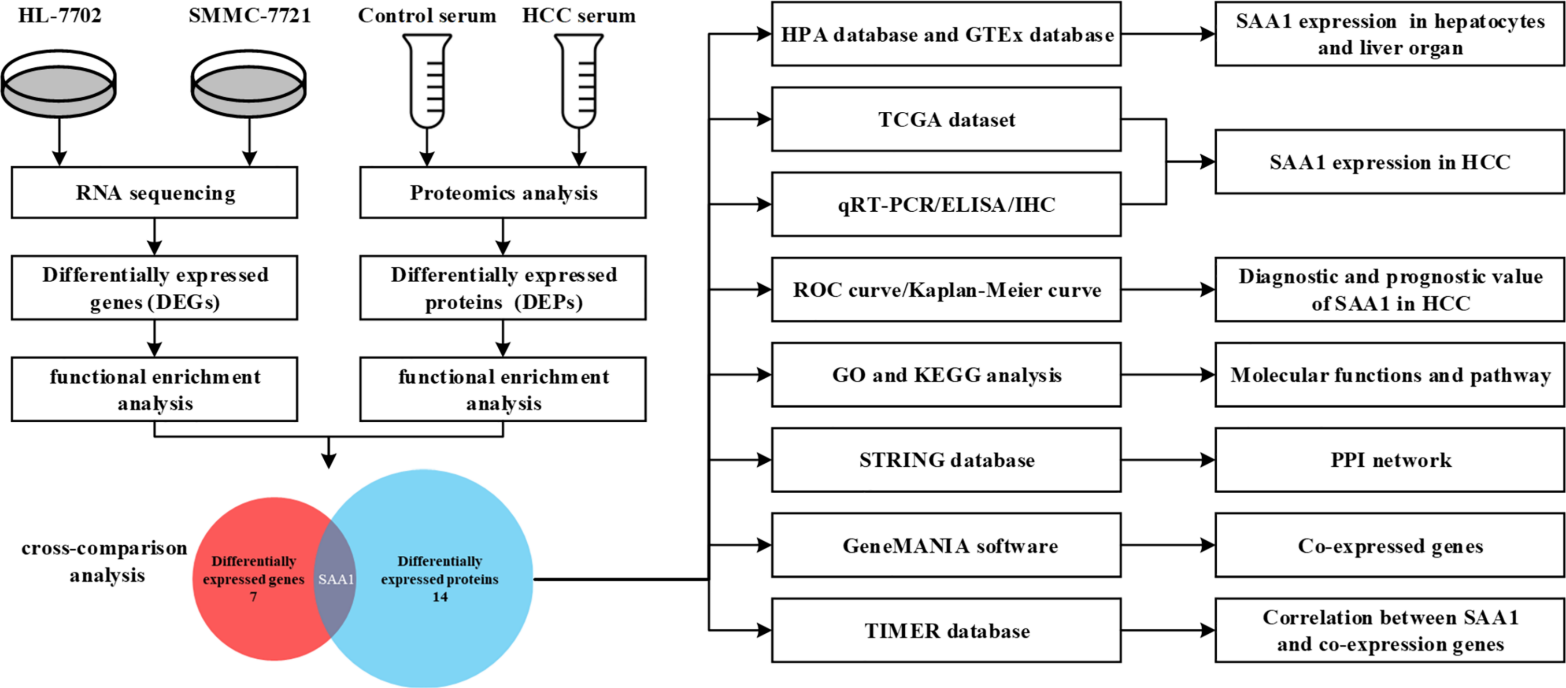
Flow chart of the study.

RNA sequencing of HCC cell line SMMC-7721 and normal liver cell line HL-7702 was performed using the Ion Proton System. On average, approximately 78.1 million 96-bp-long sequencing reads and 7.5 G of raw sequence data were obtained for samples sequenced on one lane. The normalized gene expression was measured as fragments per kilobase of transcript per million mapped reads (FPKM). To evaluate differential gene expression, the absolute value of log2-transformed fold change (FC) ≥ 1 and q-values < 0.05 were used as the criteria to determine the significance of gene expression differences. A total of 610 differentially expressed genes (DEGs) were revealed in the transcriptome comparison, 313 of which were up-regulated and 297 of which were down-regulated in HCC (**Supplemental Table 1**). Serum samples of 10 HCC patients and 10 age- and gender-matched healthy controls were selected and subjected to proteomic analysis. As a result, a total of 3541 proteins were identified and 189 differentially expressed proteins (DEPs) were observed in HCC compared with healthy controls (|FC| > 2, *P* < 0.05). The analysis process was shown in **Figure 1**.

### 3.2 SAA1 listed as an HCC metastasis candidate biomarker by cross-comparison of transcriptomic and proteomic data

**Figure 2:**
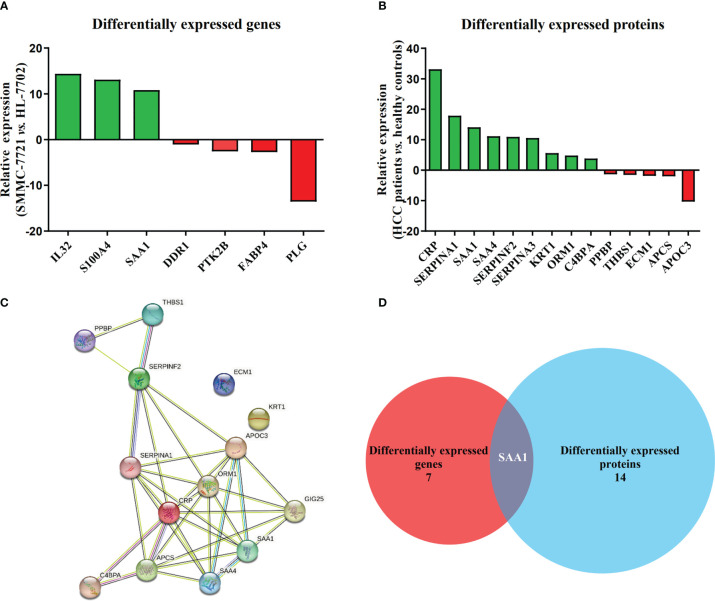
SAA1 listed as an HCC metastasis candidate biomarker. **(A)** Relative expression of 7 DEGs associated with HCC metastasis in HCC cell line SMMC-7721, when compared with normal liver cell line HL-7702. **(B)** Relative expression of 14 DEPs associated with HCC metastasis in HCC patient serums (n = 10), when compared with healthy controls (n = 10). **(C)** Constructed protein–protein interaction (PPI) network of DEPs related to HCC metastasis using Search Tool for Retrieval of Interacting Genes/Proteins (STRING) database. **(D)** SAA1 was listed as common key gene by the Venn diagram analysis.

Among all DEGs above, we found that 7 DEGs were related to HCC metastasis by functional enrichment analysis (**Table 3**), among which 3 DEGs were significantly up-regulated and 4 DGEs were significantly down-regulated (**Figure 2A**). Functional enrichment analysis found that there were 14 DEPs in HCC samples compared with healthy controls (**Table 4**), among which 9 DEPs were significantly up-regulated and 5 DGPs were significantly down-regulated (**Figure 2B**). The PPI network diagram of DEPs was constructed through STRING database (**Figure 2C**). The network contained 14 nodes and 34 edges. Then, a cross-comparison analysis was performed between transcriptomic and proteomic data. As shown in the Venn diagram (**Figure 2D**), SAA1 as common key gene was listed as a candidate biomarker of HCC metastasis for further verification.

**Table 3:** Differentially expressed genes (DEGs) associated with HCC metastasis.

No.	Gene Symbol	Regulation	SMMC-7721 *vs.* HL-7702		Gene Description
			Log2 FC	*q* Value	
1	IL32	up	14.358	0.0002	Interleukin-32
2	S100A4	up	13.096	0.0000	Protein S100-A4
3	SAA1	up	10.819	0.0001	Serum amyloid A-1 protein
4	DDR1	down	-1.060	0.0000	Epithelial discoidin domain-containing receptor 1
5	PTK2B	down	-2.558	0.0001	Protein-tyrosine kinase 2-beta
6	FABP4	down	-2.737	0.0004	Fatty acid-binding protein
7	PLG	down	-13.551	0.0005	Plasminogen

**Table 4:** Differentially expressed proteins (DEPs) associated with HCC metastasis.

No.	Protein Name	Regulation	HCC patients *vs.* Healthy controls		Protein Description
			116/113 ^1^	*p* Value	
1	CRP	up	33.1131	0.0003	C-reactive protein
2	SERPINA1	up	17.8649	0.0255	Alpha-1-antitrypsin
3	SAA1	up	14.0605	0.0002	Serum amyloid A-1 protein
4	SAA4	up	11.1272	0.0165	Serum amyloid A-4 protein
5	SERPINF2	up	10.9376	0.0152	Alpha-2-antiplasmin
6	SERPINA3	up	10.5682	0.0005	Alpha-1-antichymotrypsin
7	KRT1	up	5.6138	0.0307	Keratin, type II cytoskeletal 1
8	ORM1	up	4.7863	0.0365	Alpha-1-acid glycoprotein 1
9	C4BPA	up	3.8019	0.0055	C4b-binding protein alpha chain
10	PPBP	down	0.7365	0.0306	Platelet basic protein
11	THBS1	down	0.6486	0.0258	Thrombospondin-1
12	ECM1	down	0.5395	0.0107	Extracellular matrix protein 1
13	APCS	down	0.4966	0.0121	Serum amyloid P-component
14	APOC3	down	0.0968	0.0203	Apolipoprotein C-III

^1^ 116/113, ratio of DEPs in HCC patients relative to healthy controls.

### 3.3 SAA1 expression enriched in hepatocytes and liver organ

**Figure 3:**
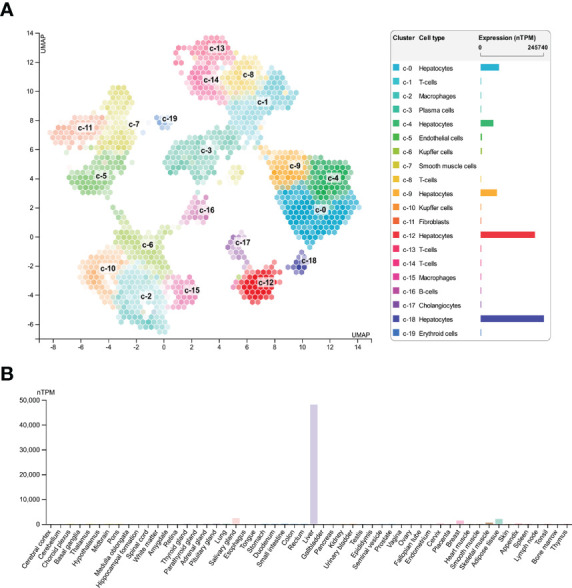
Expression of SAA1 in various cell types and organs. **(A)** SAA1 was highly enriched in hepatocytes among various cell types based on Human Protein Atlas (HPA) database. **(B)** SAA1 was also highly enriched in liver organ compared to other organs in Consensus dataset which combining the HPA and Genotype-Tissue Expression (GTEx) datasets.

To explore expression of SAA1 among various cell types and organs, we used HPA database and GTEx database to assess SAA1 expression. HPA database confirmed that SAA1 was highly enriched in hepatocytes among various cell types (**Figure 3A**). We also found that SAA1 was highly enriched in liver organ compared to other organs in Consensus dataset, which combined HPA and GTEx datasets (**Figure 3B**).

### 3.4 SAA1 was highly expressed in HCC

**Figure 4:**
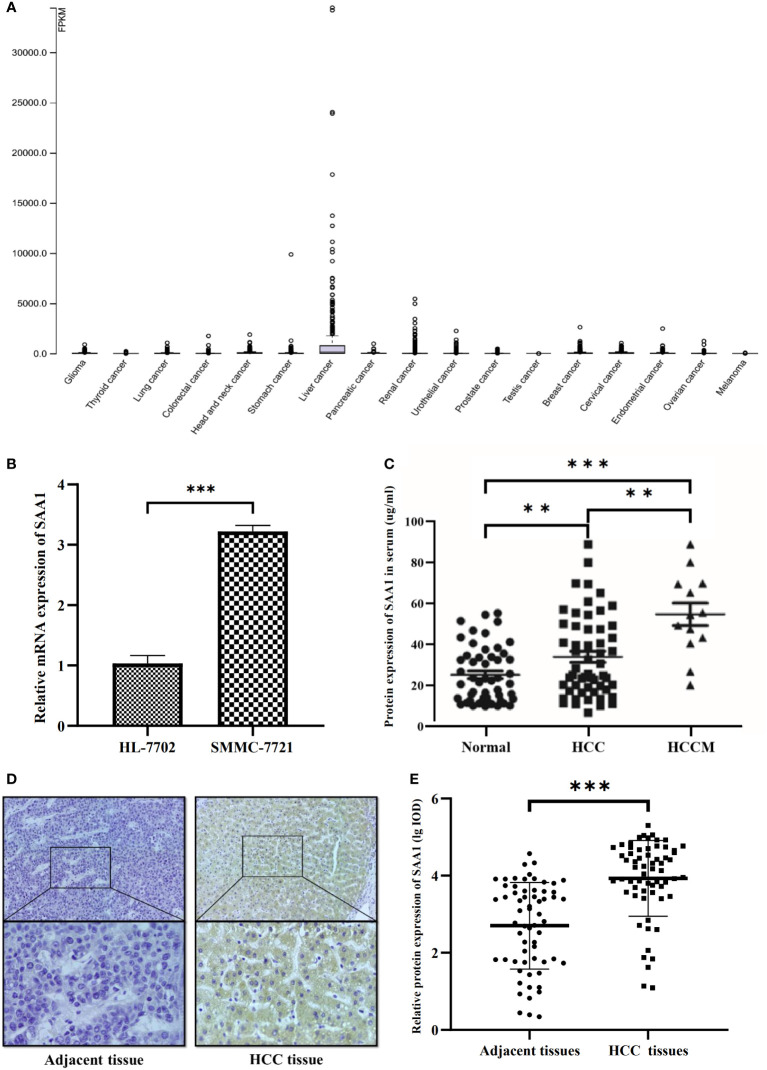
Expression of SAA1 in HCC. **(A)** SAA1 was highly expressed in liver cancer among various cancer category based on The Cancer Genome Atlas (TCGA) dataset. **(B)** SAA1 mRNA expression in SMMC-7721 cells and HL-7702 cells (*P* < 0.001). **(C)** SAA1 concentrations in serum were verified in 52 healthy controls, 55 HCC patients and 13 HCC patients with metastasis (HCCM) using ELISA (**P < 0.01, ***P < 0.001). **(D)** Immunohistochemical staining for SAA1 expression in HCC and adjacent non-tumorous liver tissues, representative images of positive staining in HCC tissues and negative staining in adjacent non-tumorous liver tissues. **(E)** Immunohistochemical staining integrated optical density (IOD) value of SAA1 in HCC and adjacent non-tumorous liver tissues (n = 63, *P* < 0.001).

To verify the reliability of the above studies, we analyzed the expression of SAA1 in The Cancer Genome Atlas (TCGA) dataset. As shown in **Figure 4A**, SAA1 was highly expressed in liver cancer among various cancer categories. Next, we analyzed the mRNA expression levels of SAA1 using qRT-PCR. The result showed that SAA1 mRNA levels in SMMC-7721 cells were 3.12 times higher than those in HL-7702 cells (*P* < 0.001) (**Figure 4B**). ELISA was also used to investigate SAA1 protein concentrations in 52 healthy controls, 55 HCC patients, and 13 HCC patients with metastasis (HCCM) serum. In consistent with the mRNA expression result, SAA1 concentrations in HCC patient serum (34.08 ± 19.89μg/mL) were significantly higher than in controls serum (25.23 ± 13.58μg/mL) (*P* < 0.01). Moreover, SAA1 concentrations in HCCM serum (53.24 ± 22.84μg/mL) were significantly higher than in HCC (*P* < 0.01) or healthy controls (*P* < 0.001) (**Figure 4C**). The protein expression of SAA1 in 63 cases of HCC and matched adjacent non-tumorous liver tissues was examined using IHC. We found that SAA1 expression presented as positive staining in HCC tissues and negative staining in adjacent non-tumorous liver tissues (**Figure 4D**). Furthermore, the expression of SAA1 protein was significantly higher in the HCC tissues than in the adjacent non-tumorous liver tissues (*P* < 0.001) (**Figure 4E**).

### 3.5 Overexpression of SAA1 predicts worse survival in HCC

**Figure 5:**
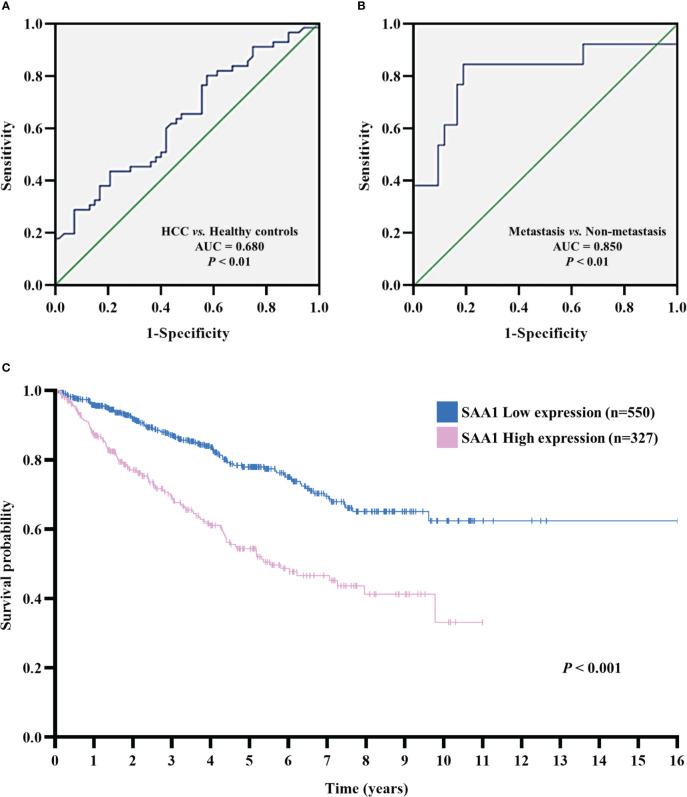
The receiver operating characteristic (ROC) and Kaplan–Meier curves analysis for SAA1 expression. **(A)** ROC curve analysis determined the predictive value of serum SAA1 expression in HCC patients and healthy controls (AUC = 0.680, *P* < 0.01). **(B)** ROC curve analysis determined the predictive value of serum SAA1 expression in HCC metastasis and non-metastasis (AUC = 0.850, *P* < 0.01). **(C)** Kaplan-Meier curve analysis of overall survival in SAA1 high expression (n = 327) and SAA1 low expression (n = 550) HCC patients based on TCGA dataset, HCC patients with a high expression of SAA1 had worse overall survival (log-rank test, *P* < 0.001).

The diagnostic and prognostic value of SAA1 in HCC were determined. We evaluated the diagnostic role of SAA1 in distinguishing between HCC patients and healthy controls through ROC curve, with AUC and critical value were 0.680 (*P* < 0.01) and 23.49μg/ml, respectively. The sensitivity and specificity of SAA1 in the diagnosis of HCC were 63.6% and 53.8%, respectively (**Figure 5A**). Next, the diagnostic role of SAA1 in distinguishing between HCC metastasis and non-metastasis was also assessed. The AUC was 0.850 (*P* < 0.01), the critical value was 40.26μg/ml, and the sensitivity and specificity were 84.6% and 81.0%, respectively (**Figure 5B**). Above ROC curve analysis results indicated that SAA1 had better predictive values for distinguishing between HCC metastasis and non-metastasis. Besides, Kaplan-Meier curve analysis revealed that HCC patients with higher SAA1 expression had worse overall survival (log-rank test, *P* < 0.001) (**Figure 5C**).

### 3.6 Analysis of co−expressed genes GO and KEGG pathways associated with SAA1 in HCC

**Figure 6:**
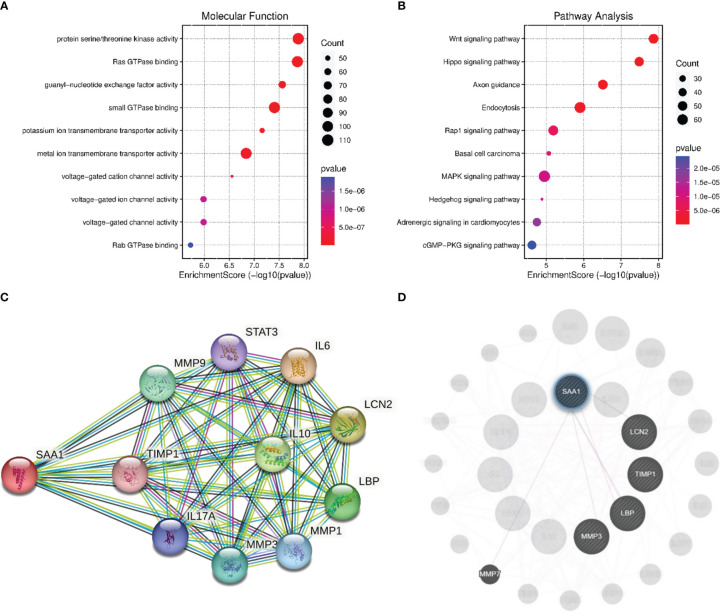
Enrichment analysis and PPI network analysis for SAA1. **(A)** The enriched molecular function items for the co-expressed items for the co-expressed genes of SAA1. **(B)** The enriched Kyoto Encyclopedia of Genes and Genomes (KEGG) items for the co-expressed items for the co-expressed genes of SAA1. **(C)** Protein network diagram of interaction with SAA1 created base on the STRING database. **(D)** Protein network diagram of interaction with SAA1 created using GeneMANIA software.

The GO analysis of gene enrichment analysis showed that the molecular functions of co−expressed genes associated with SAA1 including protein serine/threonine kinase activity, Ras GTPase binding and guanyl-nucleotide exchange factor activity (**Figure 6A**). KEGG pathway analysis showed that SAA1 may play a role in HCC by participating in Wnt signaling pathway, Hippo signaling pathway, and Rap1 signaling pathway (**Figure 6B**). STRING database was used to complete an online analysis of the SAA1 protein, producing a network diagram of the PPI between 10 genes and SSA1 gene (**Figure 6C**). Besides, we constructed PPI network for SAA1, as well as co−expressed genes by using the GeneMANIA software (**Figure 6D**). The PPI network indicated that SSA1 was co-expressed with MMP1, MMP3, and MMP9; together, they played an important role in tumor invasion and metastasis (27).

### 3.7 Correlation between SAA1 expression and co-expression genes in HCC

**Figure 7:**
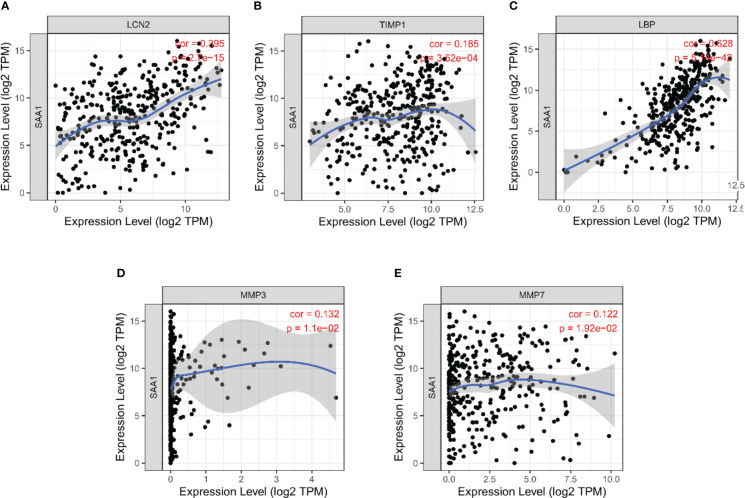
Correlation analysis between SAA1 expression level and co-expression genes in HCC based on Tumor Immune Estimation Resource (TIMER) database. **(A)** Correlation between SAA1 and LCN2 (*r* = 0.395; *P* < 0.001). **(B)** Correlation between SAA1 and TIMP1 (*r* = 0.185; *P* < 0.001). **(C)** Correlation between SAA1 and LBP (*r* = 628; *P* < 0.001). **(D)** Correlation between SAA1 and MMP3 (*r* = 0.132; *P* < 0.05). **(E)** Correlation between SAA1 and MMP7 (*r* = 0.122; *P* < 0.05).

We further analyzed the correlation between the expression level of SAA1 and co-expression genes in HCC using the TIMER database. The results showed that the expression level of SAA1 was positively correlated with LCN2 (*r* = 0.395; *P* < 0.001) (**Figure 7A**), TIMP1 (*r* = 0.185; *P* < 0.001) (**Figure 7B**), LBP (*r* = 628; *P* < 0.001) (**Figure 7C**), MMP3 (*r* = 0.132; *P* < 0.05) (**Figure 7D**), and MMP7 (*r* = 0.122; *P* < 0.05) (**Figure 7E**).

## 4 Discussion

In several developing countries, the incidence of HCC is the highest among all cancer types, and it is progressively increasing in the developed world (28). Although some new drugs are approved for treating HCC, due to metastasis and multidrug resistance, the 5-year survival rate of HCC is not significantly improved (29). Thus, it is important to identify predictive biomarkers of the pre-metastatic state and understand the molecular pathology of metastasis initiation for early diagnosis or early prevention. Over the past decade, with the advent of multi-omics technologies, the “omics”-based methods have been widely used in identifying valuable novel targets for HCC (30, 31).

In this study, the cells transcriptomic sequencing data and serum proteomics data were analyzed together to screen key genes related to HCC metastasis. We found that SAA1 is a common key candidate biomarker and highly enriched in hepatocytes and liver. It is highly expressed in HCC cells and the serum and tissues of HCC patient based on HPA, GTEx, and TCGA datasets, and *vitro* experiments. Subsequently, the prognostic value of SAA1 was confirmed to further evaluate its possible clinical applications. ROC curve analysis results indicated that SAA1 had better predictive values for distinguishing between HCC metastasis and non-metastasis. Kaplan-Meier curve analysis revealed that HCC patients with higher SAA1 expression had worse overall survival. Furthermore, GO and KEGG enrichment analysis suggested that SAA1 may involve in tumor-related pathways and co-expressed with tumor metastasis-related proteins. Thus, SAA1 may serve as a potential biomarker for HCC metastasis.

SAA1 is a member of the apolipoprotein serum amyloid A (SAA) family, and it is an acute phase protein secreted by the liver in response to inflammation, trauma, surgery or advanced malignant tumors (32). SAA1 has been reported to be a risk and prognosis biomarker in tumorigenesis, metastasis, and therapy. SAA1 is highly expressed in lung cancer, gastric cancer, endometrial cancer, prostate cancer, melanoma, and esophagus cancer, which presented poor prognosis in patients (12, 13, 15, 19, 33, 34). In addition, a large number of studies have reported that SAA1 promotes tumor progression and accelerates distant metastasis (35, 36). For example, previous studies reported that SAA1 possessed the potential to become a prognostic marker of RCC (37, 38). Serum SAA1 has been identified as a biomarker of distant metastases, but it’s not an early tumor marker in RCC patients (39). Moreover, previous study showed that SAA1 promotes tumor metastasis by inducing epithelial-mesenchymal transition in oral cancer (40). Esophageal squamous cell carcinoma with high SAA1 expression were highly aggressive and closely associated with distant metastasis of esophageal squamous cell carcinoma (41). Furthermore, SAA1 promotes αVβ3-mediated cell migration and invasion in GBM and activates the Erk signaling pathway (35). However, the role of SAA1 in HCC has never been reported.

We did not investigate the detailed mechanism by which SAA1 promoted metastasis capability in HCC. However, it has been reported that SAA1 could activate metastasis-related signaling pathways in other tumors, such as by activating the transcriptional factor nuclear factor kappa B (NF‐κB), TGF-β and AKT pathways. The NF‐κB and AKT signaling pathways have been reported to play a key role in HCC cell invasion and metastasis by promoting the expression of MMPs, which is a key factor in HCC invasion and metastasis (42–45). It has recently been reported that SAA1 knockdown in pancreatic cancer cells led to enhanced migration/invasion, drug resistance, and EMT phenotype *via* NF‐κB activation (46). Furthermore, it has been reported that SAA1 promotes Th17 cell differentiation through independently of TGF-β signaling pathway and promote inflammatory disease (47). TGF-β signaling pathway is the predominant cytokine signaling pathway in the development and progression of HCC, the imbalance of TGF-β1/BMP-7 (member of the TGF-β superfamily) pathways play key role in HCC invasion and metastasis (48). In addition, the SAA1 promotes ovarian cancer cell migration by regulating MMPs and EMT which may correlate with AKT pathway activation (49). Interestingly, we found that the expression level of SAA1 was also positively correlated with MMPs (MMP3 and MMP7) in our study. Based on the findings from literature and our results, we suggest that the invasion and metastasis effect of SAA1 in HCC may occur through the activation of NF‐κB, TGF-β and AKT signaling pathways and promotion of MMPs expression.

In summary, for the first time, we have demonstrated that SAA1 expression is significantly up-regulated at the mRNA and protein levels in HCC, and it may be associated with HCC metastasis. Moreover, SAA1 has a great potential to be the prediction biomarker of HCC metastasis.

## Data availability statement

The datasets presented in this study can be found in online repositories. The names of the repository/repositories and accession number(s) can be found in the article/**Supplementary Material**.

## Ethics statement

The studies involving human participants were reviewed and approved by The Ethics Committee of Guangxi Medical University. The patients/participants provided their written informed consent to participate in this study.

## Author contributions

Conceptualization: MH. Methodology: MH and GL. Software: GL, QS and HX. Validation: QS, HX, YZ and CL. Formal analysis: GL, QS and YL. Investigation: GL and YZ. Resources: YZ. Data curation: YZ, YL and CL. Writing-original draft preparation: GL and QS. Writing-review and editing: GL and YL. Visualization: GL, QS and HX. Supervision: MH and GL. Project administration: MH. Funding acquisition: GL and MH. All authors contributed to the article and approved the submitted version.

## Supplementary material

Supplementary Figure 1 Schematic flow of transcriptomic sequencing.

Supplementary Figure 2 Flow chart of iTRAQ-MALDI-MS/MS analysis.
