# Supplementary figures and images for: SAA1 identified as a potential prediction biomarker for metastasis of hepatocellular carcinoma via multi-omics approaches
Source: Front Oncol. 2023 Apr 4;13:1138995. doi: 10.3389/fonc.2023.1138995 (PMC10110885; doi:10.3389/fonc.2023.1138995)

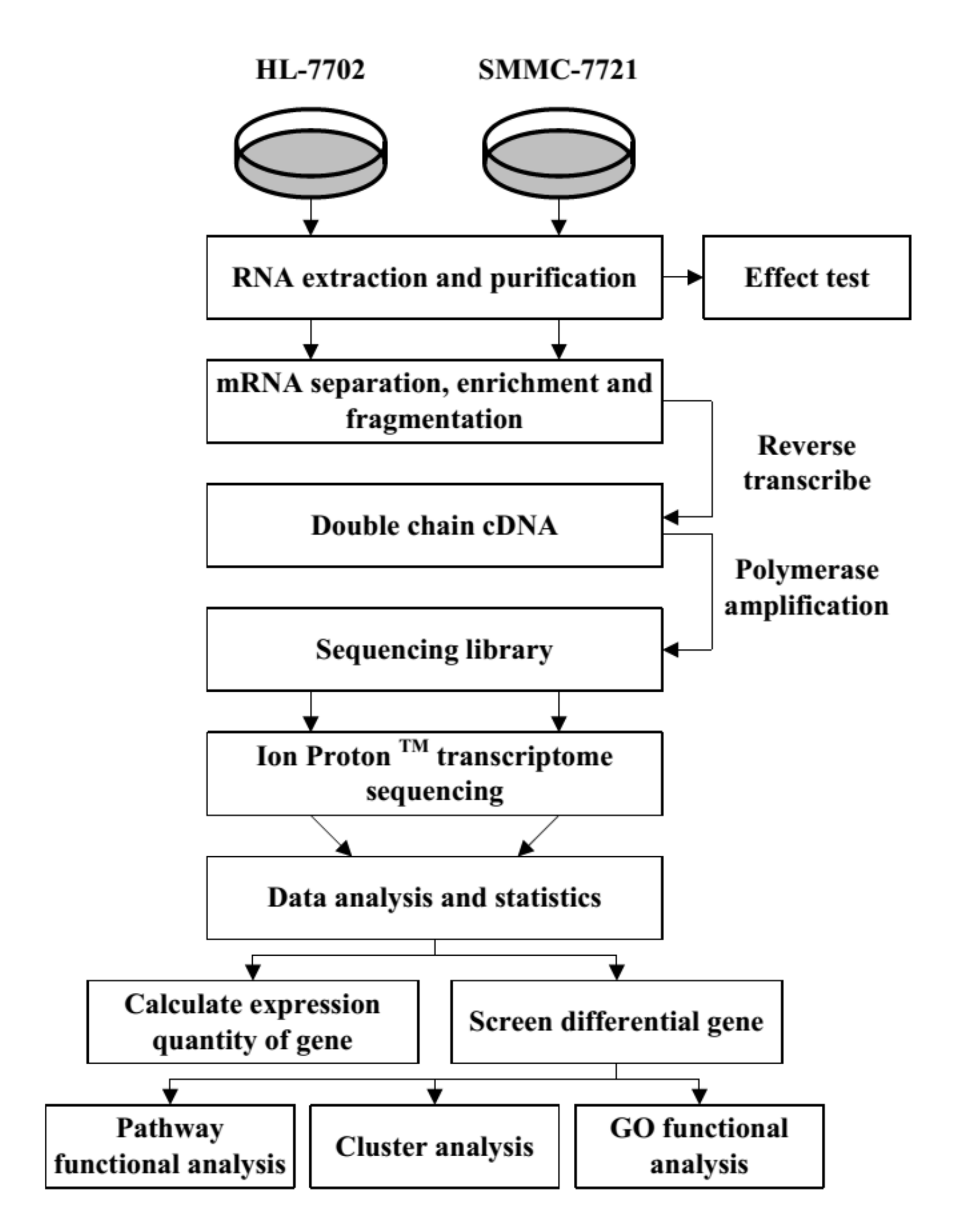

Supplement: Supplementary Figure 1 — Schematic flow of transcriptomic sequencing. [file Image_1.tiff]

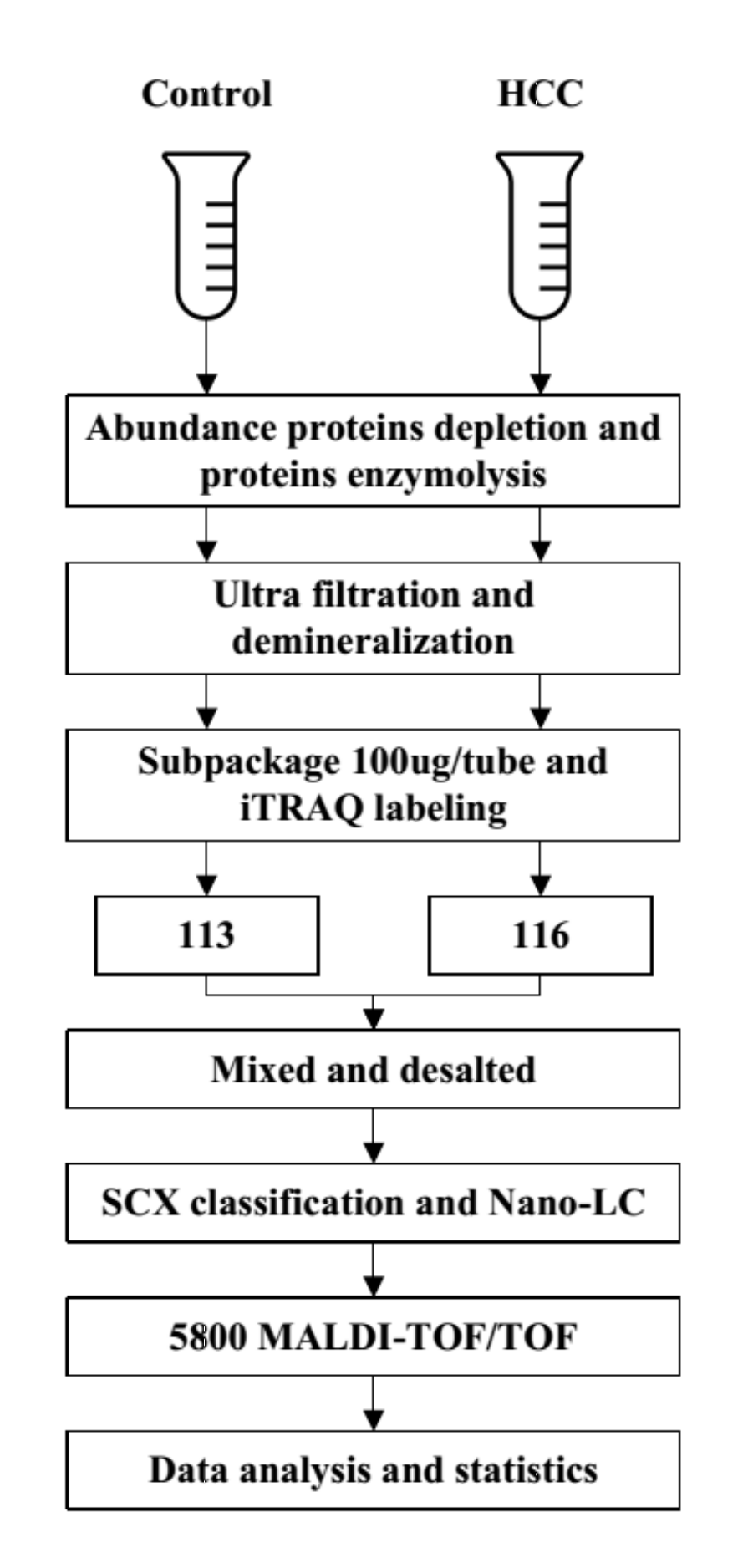

Supplement: Supplementary Figure 2 — Flow chart of iTRAQ-MALDI-MS/MS analysis. [file Image_2.tiff]
